# Preclinical study of LMP1-RNAi-based anti-tumor therapy in EBV-positive nasopharyngeal carcinoma

**DOI:** 10.1590/1414-431X2023e12638

**Published:** 2023-07-21

**Authors:** Qi Yuan, Bing-Hong Chen, Dai-jia Huang, Rong Zhang

**Affiliations:** 1 State Key Laboratory of Oncology in South China, Guangdong Key Laboratory of Nasopharyngeal Carcinoma Diagnosis and Therapy, Sun Yat-sen University Cancer Center, Guangzhou, China; 2 Department of Endoscopy, State Key Laboratory of Oncology in South China, Collaborative Innovation Center for Cancer Medicine, Sun Yat-sen University Cancer Center, Guangzhou, China; 3 Department of Endoscopy, National Cancer Center/National Clinical Research Center for Cancer/Cancer Hospital & Shenzhen Hospital, Chinese Academy of Medical Sciences and Peking Union Medical College, Shenzhen, China

**Keywords:** LMP1, ShRNAs, Anti-tumor immunity, Nasopharyngeal carcinoma, Preclinical study

## Abstract

RNA interference (RNAi) treatment has been proven to be an important therapeutic approach in cancer based on downregulation of target-oncogenes, but its clinical efficacy still needs further investigation. LMP1 is usually presented by Epstein-Barr virus (EBV)-positive tumor cells like EBV-associated nasopharyngeal carcinoma (NPC) and acts as an oncogene in tumorigenesis. However, the mechanism of LMP1 as a proto-oncogene in nasopharyngeal carcinoma is still unclear. Two sequence-specific shRNAs 1 and 2 were designed to target the different nucleotide loci of EBV latent antigen LMP1 gene and a series of *in vivo* and *in vitro* experiments were performed to investigate the therapeutic effect of sequence-specific shRNAs targeting LMP1 and its related molecular mechanisms in EBV-positive NPC. LMP1-shRNA2 generated a truncated LMP1 mRNA and protein, whereas LMP1-shRNA1 completely blocked LMP1 mRNA and protein expression. Both LMP1-shRNAs inhibited the proliferation and migration of NPC cells overexpressing LMP1 (NPC-LMP1) as well as the NPC-associated myeloid-derived suppressor cell (MDSC) expansion *in vitro*. However, LMP1-shRNA2 maintained the immunogenicity of NPC-LMP1 cells, which provoked MHC-class I-dependent T cell recognition. LMP1-shRNAs inhibited tumor growth in nude mice but did not reach statistical significance compared to control groups, while the LDH nanoparticle loaded LMP1-shRNAs and the antigen-specific T cells induced by NPC-LMP1 cells treated with LMP1-shRNA2 significantly reduced tumor growth *in vivo*. LMP1-RNAi-based anti-tumor therapy could be a new hope for the clinical efficacy of RNAi treatment of tumors like NPC.

## Introduction

RNA-based treatments are potentially superior to conventional ones, as they have a diverse target range with enhanced drug-like properties for cancer therapies (1,2). Several approaches have been employed to modulate gene-function at the RNA level in cancer cells, including base editing, small molecules targeting RNA, synthetic antisense oligonucleotides (ASOs), and exogenously expressed mRNAs (3-5). Craig C. Mello reported the role of double-stranded RNAs in post-transcriptional gene silencing through a mechanism known as RNA interference (RNAi) and revolutionized the field of gene silencing (6). In 2018, the U.S. Food and Drug Administration (FDA) approved the liver-acting siRNA ONPATTRO (patisiran) for the treatment of hereditary amyloid thyroid hormone (hATTR) with polyneuropathy, introducing RNAi drugs into the medical field (7), and provided a strong rationale to explore RNA moieties as a novel therapeutic strategy for cancers.

Short hairpin RNAs (shRNAs) can block protein expression by inducing degradation of targeted mRNAs, leading to a decrease in their levels and an inhibitory effect (8). shRNAs have opened new avenues for oncology therapy in recent years. One study showed that silencing the expression of receptor genes related to immunosuppression by shRNAs could enhance the antitumor activity and effect of CAR-T cells (9). Sequence-specific shRNAs were designed according to the characteristics of different proto-oncogenes based on the structure of shRNAs, so that they could block the action of proto-oncogenes but retain immunogenicity to stimulate the anti-tumor immunity of the host (10,11). Previous research reported that shRNA targeting a downstream loci of the dominant cytotoxic T-lymphocyte (CTL) epitope of the human papillomavirus (HPV) 16 oncogene E7 stimulates immune responses to E7-expressing tumors in C57BL/6 mice, which leads to the elimination of tumor growth *in vivo*, whereas the shRNA targeting upstream loci of CTL epitope of E7 does not (12). However, the safe delivery with a non-viral vector *in vivo* and the effectiveness of RNAi therapy with a plasmid-shRNA are largely unclear.

Latent membrane protein 1 (LMP1), an Epstein-Barr virus (EBV)-encoded primary oncogene, is a key effector molecule in undifferentiated nasopharyngeal carcinoma (NPC) pathogenesis (13). At present, LMP1 has been shown to engage in several signaling pathways that exert profound effects on the behavior of epithelial cells, such as proliferation, survival, motility, and invasion (14,15). On the one hand, it is reported that LMP1 is a participant in the immune escape of NPC by inducing myeloid-derived suppressor cell (MDSC) expansion and CTL dysfunction (16,17). On the other hand, LMP1 antigen presented by NPC cells could promote the host immune system (18,19). Based on these findings, we aimed to explore the oncogenic blocking and immunogenicity maintenance in sequence-specific LMP1-shRNAs and its clinical implication.

Therefore, we designed sequence-specific shRNAs to target the different nucleotide loci of LMP1 and performed a series of *in vivo* and *in vitro* experiments to investigate the therapeutic effect of sequence-specific shRNAs targeting LMP1 and its related molecular mechanisms in EBV-positive NPC.

## Material and Methods

The protocol of this research was approved by the Committee of Ethical Research from Sun Yat-sen University Cancer Center (Protocol Number L102032020110G).

### Cell lines

The human nasopharyngeal carcinoma cell lines NPC-TW03 (RRID: CVCL_6010) and NPC-HNE-1 (RRID: CVCL_0308) and human embryonic kidney cell line 293T were maintained in our laboratory and cultured in RPMI 1640 medium or DMEM (Life Technologies, China) supplemented with 10% fetal bovine serum (Gibco, USA). Human peripheral blood mononuclear cells (PBMCs) were isolated from the blood of healthy donors and separated by Ficoll-Hypaque gradient centrifugation method.

### Establishment and identification of LMP1 stable expression cell line

The pcDNA3.1-LMP1 expression plasmid was constructed by cloning the entire EBV-LMP1 coding sequence into the pcDNA3.1 vector for transient expression. The EBV-LMP1 coding sequence was also inserted into a lentivirus vector, FG-EH-Flag-Dest. For lentiviral production, 293T cells were transfected with the indicated vectors (δ8.9:VSVG:expression vector, 3:2:5) for 48 h, and the supernatants containing lentiviral particles were collected and stored at 4°C prior to use in the establishment of the cell lines. For the generation of the stable LMP1-expressing NPC cell lines TW03-LMP1 and HNE1-LMP1, the NPC cell lines were infected with recombinant lentivirus-transducing units plus 8 μg/mL Polybrene (Abbott Laboratories Corp., USA) and incubated for 16 h at 37°C. At 48 h after infection, the medium was replaced with fresh medium containing 1 μg/mL geneticin sulfate and cultured for another 7 days to establish NPC cell lines stably expressing LMP1. Immunoblot, cell growth, and migration *in vitro* assays were performed to determine the expression of LMP1 (Supplementary Figure S1A-D) as described previously (20).

### Design of shRNA

LMP1 was amplified and sequenced. shRNAs were designed according to the cDNA sequenced. We used the website (http://rnaidesigner.lifetechnologies.com/rnaiexpress/) to design shRNA based on the LMP1 sequence. The restriction endonuclease sites AgeI and EcoRI of PLKO.1 vector were used. The recombinant vector was constructed by combining Oligo dilution with the linearization vector under the action of T4 ligase. Competitive cells were added and the mix was applied to a flat plate, and monoclonal colonies were selected. After sequencing, the plasmid was amplified. The sequences of the shRNA targeting LMP1 are as follows:

LMP1-shRNA1: 5′:GATCCGGCTGTACATCGTTATGAGTTTTCAAGAGACTCATAACGATGTACAGCTTTTTTTTGGAATT and 3′:CTAGGCCGACATGTAGCAATACTCAAAAGTTCTCTGAGTATTGCTACATGTCGAAAAAAAACCTTAA; LMP1-shRNA2: 5′:GATCCGCCAGTTCAGCTAAGCTACTTTTCAAGAGAGTAGCTTAGCTGAACTGGTTTTTTTTGGAATT and 3′:CTAGGCGGTCAAGTCGATTCGATGAAAAGTTCTCTCATCGAATCGACTTGACCAAAAAAAACCTTAA.

### shRNA transfection

**Figure 1:**
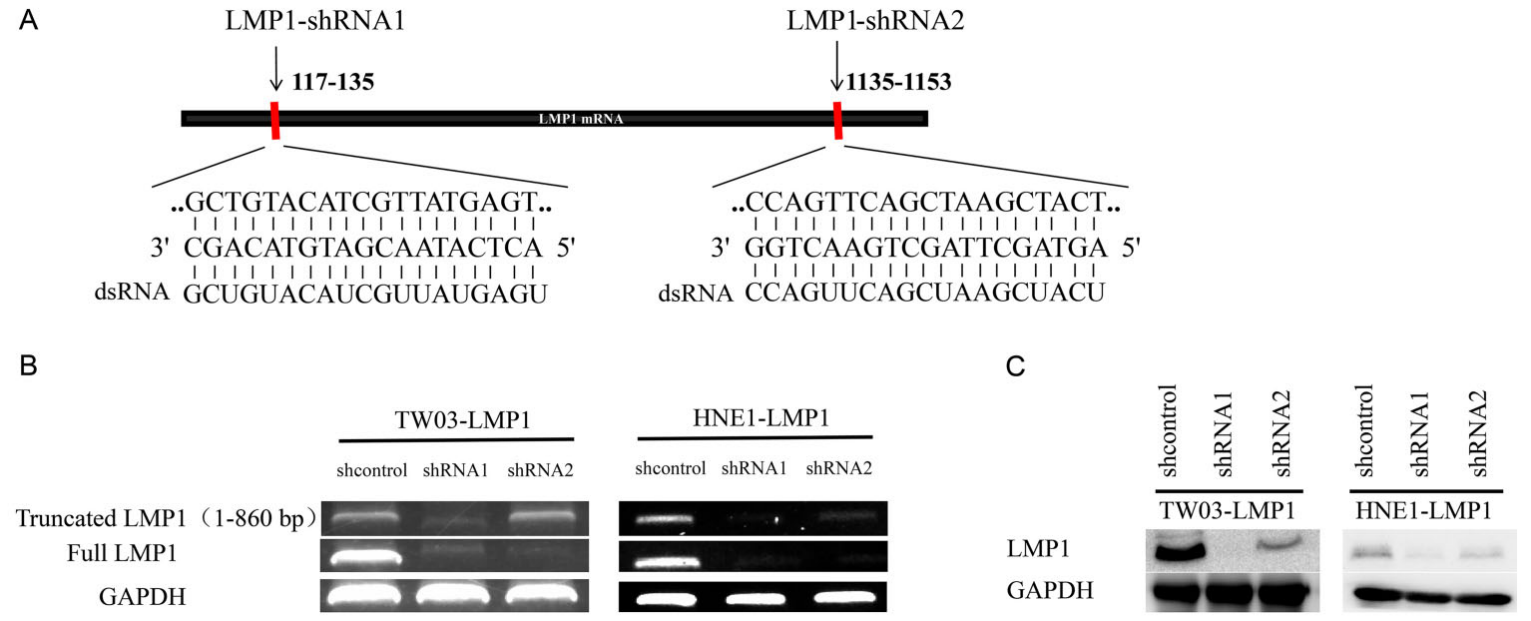
Design of sequence-specific shRNAs targeting LMP1. TW03-LMP1 and HNE1-LMP1 cells were treated with sequence-specific shRNAs targeting LMP1. **A**, Diagrammatic representation of the mRNA encoding full-length LMP1 sequence (CDS). The shRNA targeting the 117-135 base pair (bp) of LMP1 CDS is named LMP1-shRNA1, while the shRNA targeting 1135-1153 bp of LMP1 CDS is named LMP1-shRNA2. **B**, mRNA expression of LMP1 in TW03-LMP1 and HNE1-LMP1 cells under the administration of lentivirus containing shcontrol, LMP1-shRNA1, and LMP1-shRNA2 sequences. **C**, LMP1 protein expression in TW03-LMP1 and HNE1-LMP1 cells after the administration of lentivirus containing shcontrol, LMP1-shRNA1, and LMP1-shRNA2 sequences. GAPDH was also assayed as the loading control.

To alter LMP1 levels in TW03-LMP1 and HNE1-LMP1 cells, LMP1-specific shRNA (LMP1-shRNA1, LMP1-shRNA2) and control shRNA (sh-control) were cloned into the pLKO.1 vector (Addgene_52920) (Figure 1A). All plasmids were confirmed by sequencing and transfected into cells by Lipofectamine 2000 (Invitrogen, USA) (21). For lentiviral production, 293T cells were transfected with the indicated vectors (δ8.9:VSVG:expression vector, 3:2:5) for 48 h, and the supernatants containing lentiviral particles were collected and stored at 4°C prior to use in the establishment of the cell lines TW03-LMP1-sh-control, TW03-LMP1-shRNA1, TW03-LMP1-shRNA2, HNE1-LMP1-sh-control, HNE1-LMP1-shRNA1, and HNE1-LMP1-shRNA2. The cell lines were selected with 2 μg/mL puromycin.

### Quantitative real-time PCR

Total RNAs from cells were extracted with TRIzol reagent (Invitrogen) according to the manufacturer's protocol, and a Revert Aid First Strand cDNA Synthesis Kit (Invitrogen) was used to reverse transcribe. Synthesized cDNAs were then used to quantify target genes with ChamQ SYBR qPCR Master Mix (Vazyme, China) using the following primers: LMP1: forward: 5′-ACTCCTACTGATGATCACCCTCCT-3′; reverse-860: 5′-TCAGTGTTGTCAGGGTCCTGAG-3′; reverse-full: 5′-CGCCAGAGCATCTCCAATAAGTAGA-3′. GAPDH: forward: 5′-GGAGCGAGATCCCTCCAAAAT-3′; reverse: 5′-GGCTGTTGTCATACTTCTCATGG-3′.

### Immunoblot assays

For immunoblot assays, cells were harvested and subjected to lysis in ice-cold low-salt lysis buffer (LSB; 150 mM NaCl, 50 mM HEPES pH 7.5, 1.5 mM MgCl_2_, 1 mM EDTA, 10% glycerol, 1% Triton X-100) supplemented with 5 mg/mL protease inhibitor cocktail (Roche, Germany). Aliquots of the extracts (20-25 μL) were loaded to SDS-PAGE, and the membranes were immunoblotted with the indicated antibodies. Antibodies targeting the following proteins were used: anti-mouse-LMP1 antibody (AB_2161795) from Bioss (China) and anti-rabbit-GAPDH antibody (AB_843142) from Proteintech (USA).

### Cell proliferation assay

Cell viability was evaluated by colorimetric assay using 3-(4,5-dimethylthiazol-2-yl)-2,5-diphenyltetrazolium bromide (MTT). Briefly, cells were seeded onto 96-well plates at a density of 1×10^4^ and allowed to adhere. After incubation for various time intervals, cells were stained with 20 µL sterile MTT dye (0.5 mg/mL, Sigma-Aldrich, USA) for 4 h at 37°C. Then, the supernatant was removed, and the resultant formazan crystals were dissolved in dimethyl sulfoxide (DMSO, Sigma-Aldrich). The absorbance value was read at 570 nm using a microplate reader.

### Wound healing assay

Cells were subcultured in 6-well plates at a density of 2×10^5^ cells/well and allowed to form a confluent monolayer. After removal of the culture medium, the cell monolayer was gently scratched with a 200-µL pipette tip to create a linear wound. The wounded monolayer was washed twice with PBS to remove cell debris, and cells were then allowed to migrate into the cell-free area. The scratch area was photographed immediately and 12 and 24 h after scratching. Cell migration was calculated as the mean percentage of the cell migrated distance compared with the initial wound distance.

### Generation of NPC-LMP1 antigen-specific T cells

Anti-CD3 antibody (Abcam, USA) was diluted into 1 μg/μL OKT3 by PBS, which was used to stimulate the cells in 24-well plate. An aliquot of 400 μL anti-CD3 was added to each well the day before the experiment at 4°C. Then OKT-3 was removed, 2 mL of culture medium (X-vivo+1000U/mL IL-2) was added to each well, and 2×10^6^ peripheral blood mononuclear cells (PBMCs) were added at a ratio of 30:1 to irradiated NPC-LMP1, NPC-LMP1-shRNA1, or NPC-LMP1-shRNA2 treated cells (PBMC:tumor cells - 30:1, the indicated tumor cells were irradiated by an irradiator with an irradiation dose of 160 Gy). The 24-well plate was placed into a cell incubator, and the irradiated tumor cells were used for second induction stimulation after 7 days of co-culture. After 14 days of short-term co-culture, T cells were collected for detection.

### Tumor-associated MDSC induction *in vitro*

Tumor-associated MDSCs were generated from CD33+cells isolated by anti-CD33 beads (Miltenyi Biotec Company, Germany) from PBMCs of healthy donors in a co-culture Transwell System (0.4 μm pore, Corning) with the NPC cell lines TW03-LMP1-sh-control, TW03-LMP1-shRNA1, or TW03-LMP1-shRNA2 as previously described (16). HLA-DR-CD11b+CD33+cells were defined as MDSCs and measured using a fluorescence-activated cell sorting (FACS) analysis.

### Fluorescence-activated cell sorting (FACS) analysis

For FACS analysis, single-cell suspensions were stained with the appropriate fluorescent antibodies according to the manufacturer's instructions. Fluorescent antibodies used to stain human cell surface markers were purchased from eBioscience (USA): HLA-A2 (AB_2561568), CD3 (AB_1283254), CD4 (AB_1283378), CD8 (AB_1283663), IFN-γ (AB_2751182), granzyme B, HLA-DR (AB_11132296), CD33 (AB_1050523), and CD11b (AB_2536484). Data were acquired with a Beckman Coulter Gallios (Beckman, USA) flow cytometer and analyzed using CytExpert (SCR_017217) software.

### Cytotoxicity measurement

Cytotoxicity measurement was analyzed by LDH (lactate dehydrogenase) Cytotoxicity Assay Kit (C0017, Beyotime Biotechnology, China). T cells specifically induced for 14 days were collected, and tumor cells were added into the 96-well plate at a certain experimental proportion for a 6-h cell killing experiment. After 6 h, the 96-well plate was gently shaken to ensure the uniform distribution of LDH in the supernatant. The 96-well plates were centrifuged at room temperature at 600 *g* for 10 min, then 10 μL of supernatant was transferred from each plate to the new 96-well plate, and 100 μL of the LDH mixture was added to each well for 30 min incubation at room temperature. Finally, the absorbance value was measured at 450 nm (reference wavelength was 650 nm) in the enzyme plate analyzer. The killing ratio was calculated as follows:

$$\text{Cytotoxicity}\%=\frac{\text{Experimental}-\text{effector spontaneous}-\text{target spontaneous}-\text{blank}}{\text{Target Maximum}-\text{Target Minimum}}\times 100$$

### HNE1-LMP1 nude mice xenograft model and treatment

Four-week-old female BALB/c nude mice were purchased from Yaokang Biological Technology Co., Ltd, China. The mice were kept in a specific-pathogen-free condition and treated in accordance with the guidelines for the use of experimental animals by the Committee on the Use of Live Animals in Teaching and Research of the Sun Yet-sen University. For injection, 5×10^6^ HNE1-LMP1 cells suspended in 100 µL PBS were subcutaneously injected (*sc*) in the back skin of the mice and the mice were randomly grouped. Layered double hydroxide (LDH) nanoparticles were synthesized and characterized as previously described (22). The typical morphology of nano-LDH was hexagonal and plate-like shape with lateral diameters from 60 to 400 nm assessed by transmission electron microscopy. For animal treatment, nano-LDH loaded with different plasmids were administered to the mice every 3 days for 3 times. The *in vivo* treatment conditions for nano-LDH were described below. First, the nano-LDH and LMP1-shRNA1, LMP1-shRNA2, or sh-control plasmids were mixed at a 10:1 mass ratio, and the mixture was slowly added to a constant volume of bovine serum albumin (BSA) solution (5 times the concentration of nano-LDH). Then, the above solution was diluted with PBS to the specified concentration and administered to the mice by caudal vein injection (Group 1: PBS, Group 2: Nano-LDH + 1.5 mg/kg sh-control plasmid, Group 3: Nano-LDH + 1.5 mg/kg LMP1-shRNA1 plasmid, Group 4: Nano-LDH + 1.5 mg/kg LMP1-shRNA2 plasmid, Group 5: 1.5 mg/kg LMP1-shRNA1 plasmid, Group 6: 1.5 mg/kg LMP1-shRNA2 plasmid). Specific inducted T cells were administered to the mice once by caudal vein injection (Group 7: 2×10^6^ CTL1 (NPC-LMP1-sh-control induced T cells), Group 8: 2×10^6^ CTL2 (NPC-LMP1-shRNA1 induced T cells), Group 9: 2×10^6^ CTL3 (NPC-LMP1-shRNA2 induced T cells). Tumor growth was monitored until 2 weeks after the last treatment. The tumor volume was calculated using the following formula: V = W^2^ × L / 2 (W: the shortest diameter, L: the longest diameter). Then, the mice were sacrificed, the tumors were dissected and weighed, and tumor tissues were harvested.

### Statistical analysis

All statistical analyses were performed using GraphPad Prism 5 (SCR_002798) software (USA) and SPSS 18.0 (SCR_002865) software (USA). The *in vitro* testing results were produced from at least three independent experiments. Numerical data are reported as mean±SD, and statistical significance was determined using one-way analysis of variance (ANOVA) among more than two groups or a standard two-tailed Student's *t-*test or paired Student's *t*-test for two groups. In this study, P<0.05 was considered significant.

## Results

### Design of sequence-specific shRNAs targeting LMP1

ShRNA, a type of small RNA for RNAi therapy, has high specificity for gene silencing and long duration (23). Here, we designed shRNA sequences specifically targeting the 117-135 nucleotide loci (LMP1-shRNA1) and 1135-1153 nucleotide loci (LMP1-shRNA2) of LMP1 gene (Figure 1A), hoping that the shRNA2 would silence the gene and enhance its immunogenicity. As a negative control, a scrambled shRNA from shRNA2 was also designed and used (sh-control) in each experiment. We found that truncated mRNA and protein products could be generated in cells treated with LMP1-shRNA2 but not LMP1-shRNA1 (Figure 1B and C). Our data indicated that these sequence-specific shRNAs could specifically target different nucleotide loci of LMP1, interfering with LMP1 gene expression in NPC cells.

### LMP1-shRNA2 treatment maintained immunogenicity of NPC-LMP1 cells and provoked T cell recognition *in vitro*

**Figure 2:**
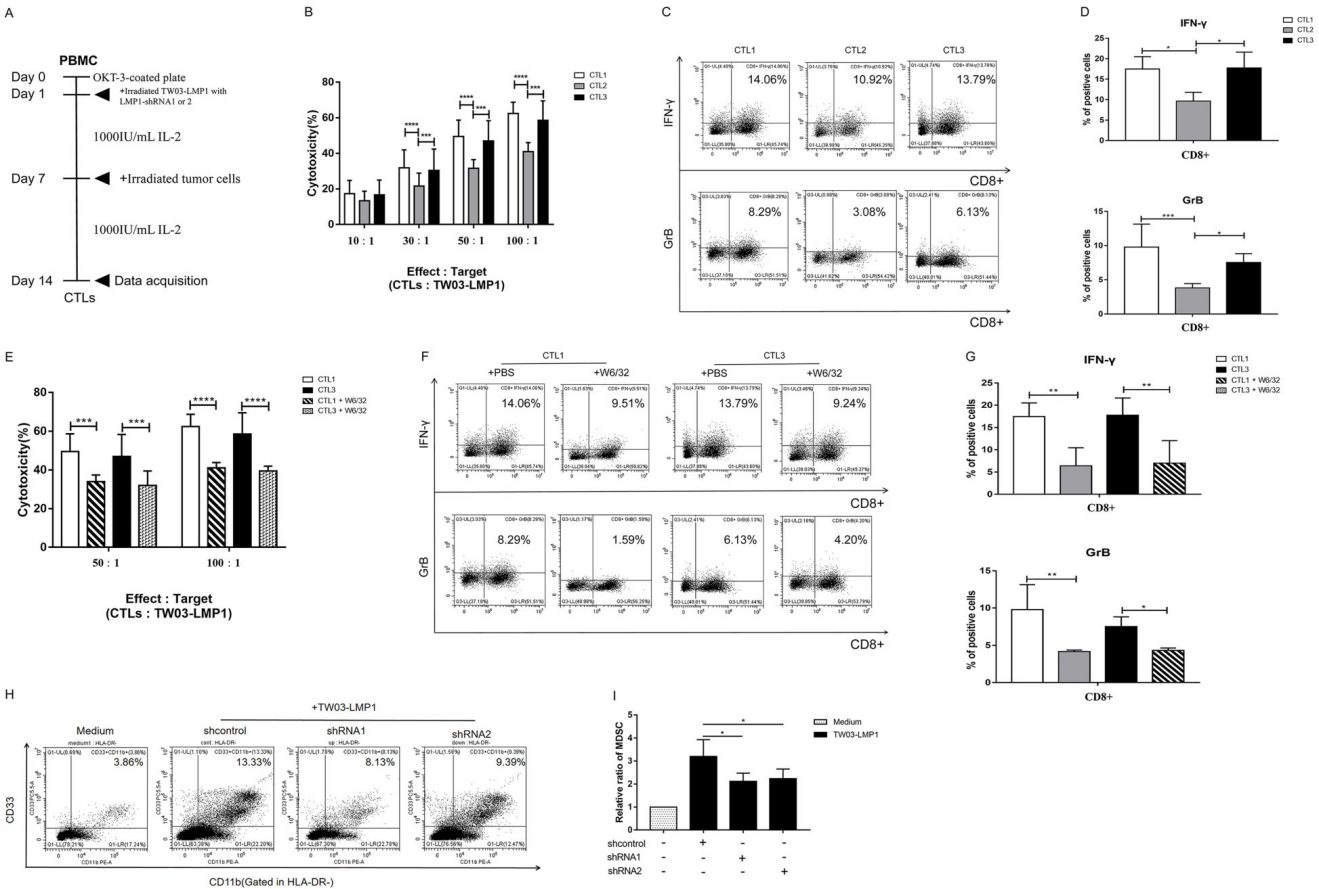
Sequence-specific shRNA targeting LMP1 retains the nasopharyngeal carcinoma (NPC)-LMP1 immunogenicity and provokes T cell immunity. **A**, Schematic illustration of the generation of NPC-LMP1-antigen-specific cytotoxic T-lymphocyte (CTLs) from peripheral blood mononuclear cells (PBMCs) of healthy donors by co-culturing with the irradiated TW03-LMP1 cells under the treatment of shcontrol, LMP1-shRNA1, and LMP1-shRNA2 in interleukin (IL)-2 medium for 14 days and re-stimulated with the irradiated TW03-LMP1 cells at day 7. **B**, *In vitro* cytotoxicity measurement of NPC-LMP1-antigen-specific CTLs induced by the above indicated condition against TW03-LMP1 cells at different ratios for 6 h. The killing capacity of CTLs was determined via LDH Cytotoxicity Assay Kit. **C** and **D**, NPC-LMP1-antigen-specific CTLs induced by the above condition co-cultured with TW03-LMP1 cells for 4 h and harvested for FACS staining. The FACS plot (**C**) and the statistical graph (**D**) show the releasing level of interferon (IFN)-γ and granzyme (GrB) from CD8+ T cells. Representative FACS plots are shown from 1 of 3 independent experiments. **E**, Cytotoxicity measurement of NPC-LMP1-antigen-specific CTLs induced by the above condition against TW03-LMP1 cells at 50:1 and 100:1 ratios in the presence of MHC-class I blocking antibody (W6/32). **F** and **G**, The NPC-LMP1-antigen-specific CTLs induced by the above condition were co-cultured with TW03-LMP1 cells in the presence of MHC-class I blocking antibody (W6/32) for 4 h and harvested for FACS staining. The FACS plot (**F**) and statistical graph (**G**) of 1 of 3 independent experiments show the level of IFN-γ and granzyme (GrB) in CD8+ T cells. **H** and **I**, CD33+cells were isolated from healthy PBMCs using human CD33 microbeads and co-cultured with TW03-LMP1 cells under the treatment of shcontrol, LMP1-shRNA1, and LMP1-shRNA2 in a Transwell system for 48 h. The percentage of myeloid-derived suppressor cells (MDSCs) was measured by FACS staining. Representative density plots are shown as the CD33+CD11b+cells in the HLA-DR cell population (**H**) and statistical graph of the percentage of MDSCs induced by NPC-LMP1 cells under the indicated treatment (**I**) are shown from at least three independent experiments. Data are reported as means±SD. *P<0.05, **P<0.01, ***P<0.001, ****P<0.0001 (Student's *t*-test).

In a previous report, siRNAs or shRNAs were used to silence HPV oncogenes E6 and E7 in cervical cancer cell lines and inhibit tumor growth (12). The authors also found that mice inoculated with shRNAs-treated tumor cells were protected from subsequent tumor challenge. We wondered whether the sequence-specific shRNA not only interfered with the oncogenic function of LMP1 but also retained LMP1 antigenicity in NPC cells. We thus established an LMP1 overexpression NPC cell line and a mimicked tumor microenvironment *in vitro* by co-culturing PBMC and tumor cells to induce NPC-LMP1 antigen specific T cells (Figure 2A), as described in the Methods section. Compared with CD8+ CTLs induced by TW03-LMP1-shRNA1 cells, the CD8+ CTLs induced by TW03-LMP1-shRNA2 cells displayed a stronger killing capability and higher level of cytokine IFN-γ and GrB releasing targeting TW03-LMP1 cells; similar results were obtained in the HNE1-LMP1 cell line (Figure 2B-D, Supplementary Figure S1E and F). The cytotoxicity and release of cytokine IFN-γ and GrB from TW03-LMP1-shRNA2 cell-induced CD8+ CTL when targeting TW03-LMP1 cells could be inhibited by MHC-class I neutralizing antibody (Figure 2E-G). Importantly, we found that both LMP1-shRNA1 and LMP1-shRNA2 treatment reduced the tumor-associated MDSC differentiation *in vitro* (Figure 2H and I). A previous report showed that LMP1 induces MDSC expansion in NPC, leading to anti-tumor immunosuppression (16). These results indicated that blocking of LMP1 by both LMP1-shRNA1 and 2 reduced the LMP1-induced immunosuppression in the NPC microenvironment, but the sequence-specific LMP1-shRNA2-mediated LMP1 blocking not only retained NPC-LMP1 cell immunogenicity but also provoked MHC-class I-dependent T cell recognition and specific cytotoxicity.

### LMP1-shRNAs inhibited NPC-LMP1 cell growth and migration *in vitro*

**Figure 3:**
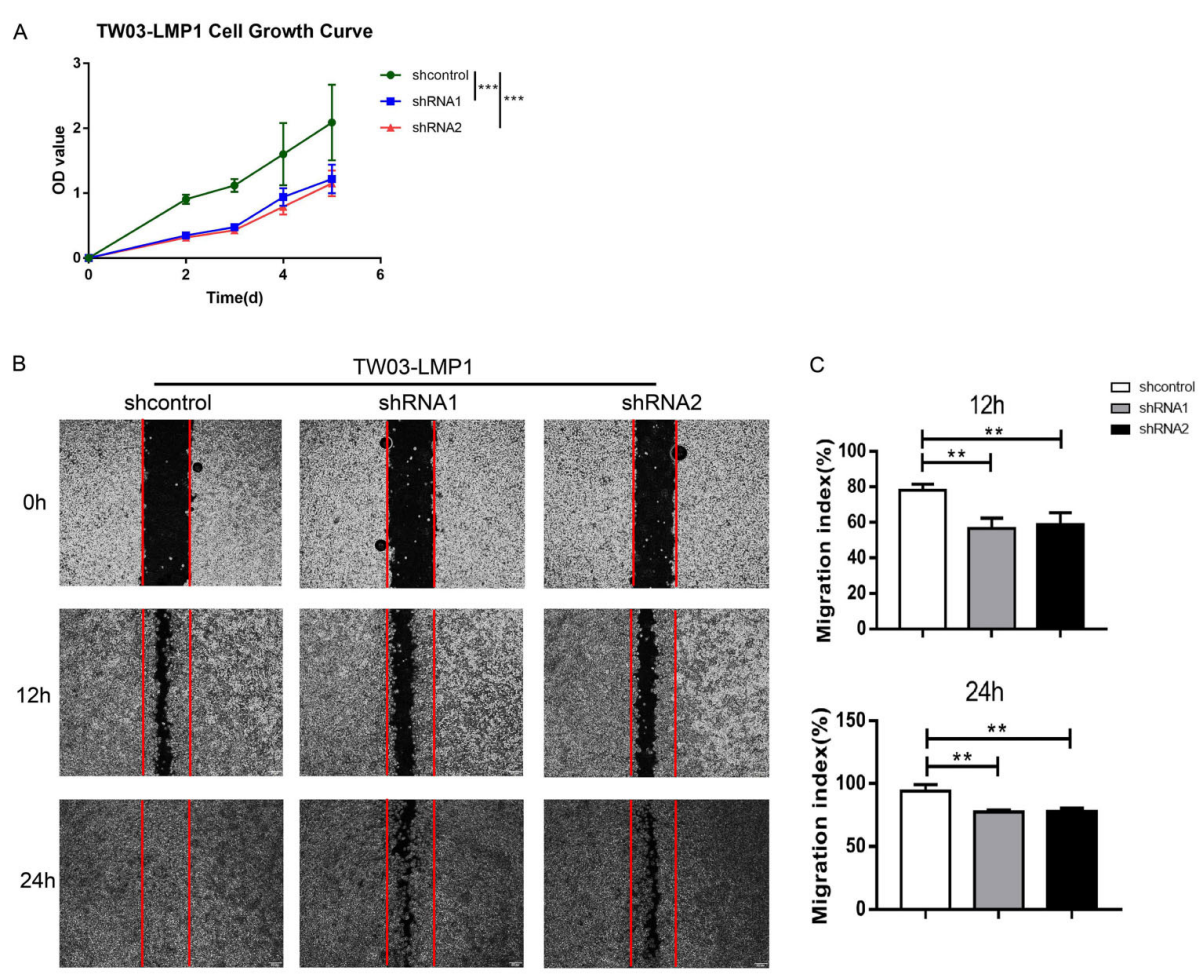
LMP1 targeting by sequence-specific shRNA inhibited nasopharyngeal carcinoma (NPC)-LMP1 cell growth and migration. **A**, Cell growth curve showing the proliferation of TW03-LMP1 cells after the treatment of shcontrol, LMP1-shRNA1, and LMP1-shRNA2, according to MTT cell proliferation assay. Statistical analysis was done with data from at least three independent experiments. **B**, Wound healing assays of TW03-LMP1-shcontrol, TW03-LMP1-shRNA1, and TW03-LMP1-shRNA2 cells. Wound closures were photographed at 0, 12, and 24 h after removing the scratched cells. **C**, Migration rates were calculated with data from at least three independent experiments. Data are reported as means±SD. **P<0.01, ***P<0.001 (Student's *t*-test).

LMP1 is a known oncogene in NPC (24). To investigate the effect on biological function of truncated LMP1 protein induced by LMP1-shRNA2, we detected NPC-LMP1 cell proliferation and migration after treatment of LMP1-shRNA1, LMP1-shRNA2, and sh-control. We found that both LMP1-shRNA1- and LMP1-shRNA2-silencing significantly decreased TW03-LMP1 cell growth compared with that of sh-control (Figure 3A). In the scratch experiment, we demonstrated that both LMP1-shRNA1- and LMP1-shRNA2-silencing reduced the TW03-LMP1 cell migration compared with that of sh-control (Figure 3B and C). These data indicated that both LMP1-shRNA1 and 2 can effectively silence LMP1 gene and reduce NPC-LMP1 cell growth and migration, and the truncated LMP1 protein generated by LMP1-shRNA2 treatment also lost the function of oncogene.

### NanoLDH-loaded LMP1-shRNAs and NPC-LMP1-specific T cells inhibited NPC-LMP1 xenograft tumor growth *in vivo*

**Figure 4:**
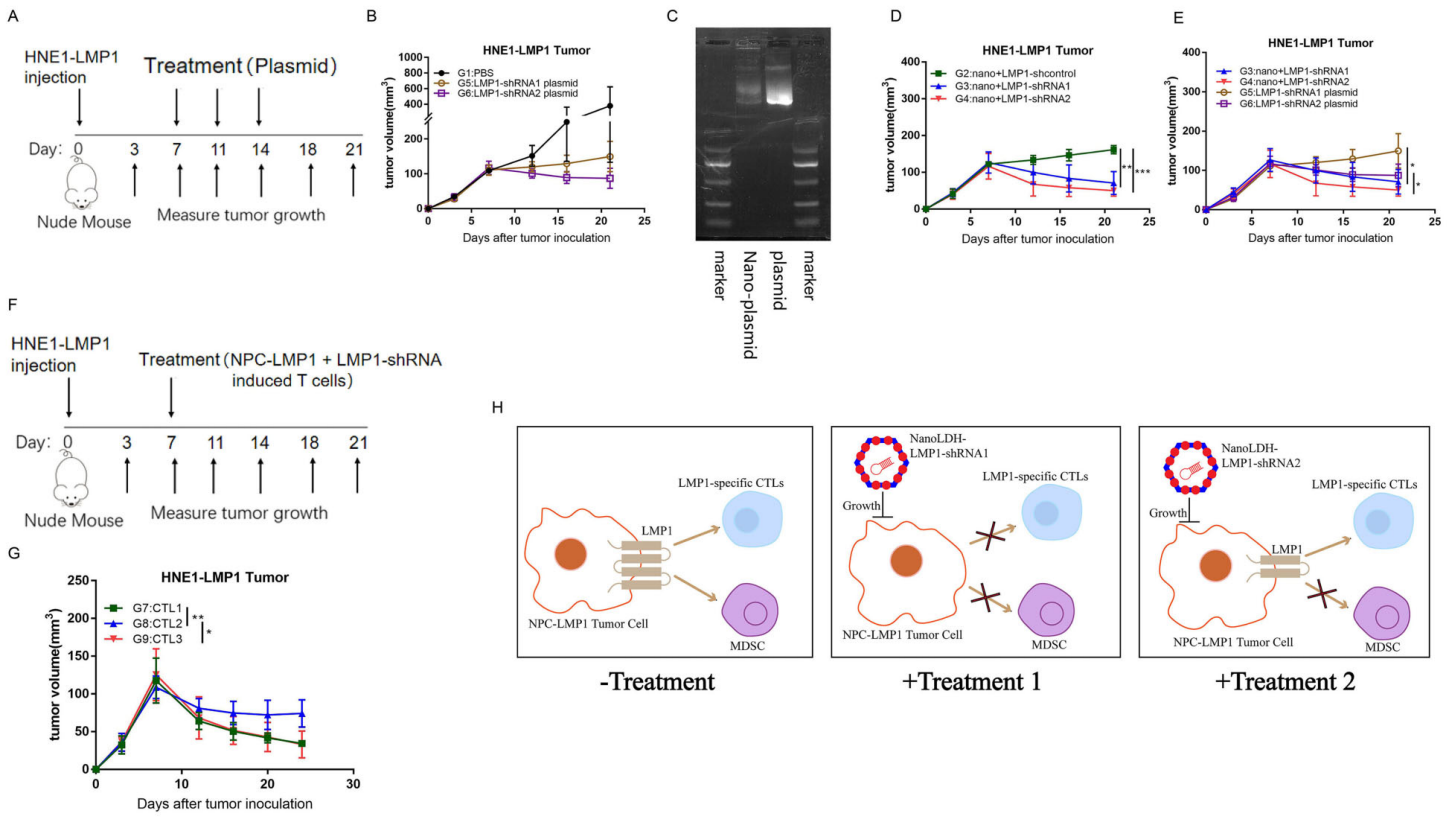
Sequence-specific shRNAs targeting LMP1 inhibited tumor growth *in vivo*. **A**, Protocol of subcutaneous injection of xenograft tumor and plasmid treatment (by intravenous injection) procedure in nude mice. **B**, Growth curves of xenograft tumor volumes of the nude mice in three groups: G1: PBS 100 µL, G5: LMP1-shRNA1 plasmid (100 µL, 1.5 mg/kg), and G6: LMP1-shRNA2 plasmid (100 µL, 1.5 mg/kg). All groups were treated by caudal vein injection. **C**, Plasmids and nanomaterials were mixed *in vitro* in accordance with the requirements of the use of nanomaterials. PCR results show the nanomaterial and plasmid binding. **D** and **E**, Growth curves of tumor volumes of the nude mice in several groups: G2: Nano-LDH + shcontrol (100 µL, 1.5 mg/kg), G3: Nano-LDH + LMP1-shRNA1 (100 µL, 1.5 mg/kg), G4: Nano-LDH + LMP1-shRNA2 (100 µL, 1.5 mg/kg), G5: LMP1-shRNA1 plasmid (100 µL, 1.5 mg/kg), and G6: LMP1-shRNA2 plasmid (100 µL, 1.5 mg/kg). All groups were treated by caudal vein injection. **F**, Protocol of subcutaneous injection of xenograft tumor following the NPC-LMP1 antigen-specific T cells treatment (by intravenous injection) procedure in nude mice. **G**, Growth curves of xenograft tumor volumes of the nude mice in each group, including G7: CTL1 (NPC-LMP1-sh-control cells induced T cells) (200 µL, 2.5*10^6^ T cells), G8: CTL2 (NPC-LMP1-shRNA1 cells induced T cells) (200 µL, 2.5*10^6^ T cells), and G9: CTL3 (NPC-LMP1-shRNA2 cells induced T cells) (200 µL, 2.5*10^6^ T cells). Data are reported as means±SD (n=3). *P<0.05, **P<0.01, ***P<0.001 (Student's *t*-test). **H**, Schematic diagram of the mechanisms of specific-sequence shRNAs targeting LMP1 and inhibiting NPC growth.

We further investigated the function of LMP1-shRNA1 and LMP1-shRNA2 on tumor growth *in vivo*. The HNE1-LMP1 cells were subcutaneously injected into nude mice to grow NPC-LMP1 xenograft tumors, and then the plasmid of LMP1-shRNA1, LMP1-shRNA2, and sh-control were injected through the caudal vein at indicated time points, as shown in Figure 4A. We found that the tumor volume was reduced in the LMP1-shRNA1 and LMP1-shRNA2 groups compared with that of the sh-control but did not reach statistical significance (Figure 4B). Based on the above data, we speculated that the instability and low cell up-taking ability of shRNAs might reduce the efficacy of shRNAs *in vivo*. Therefore, we employed a nanoparticle delivery system by loading the shRNA plasmid to the nano-LDH *in vitro* in accordance with the required ratio of the nanomaterials, which was verified via PCR assessment (Figure 4C). Then, we treated the HNE1-LMP1 tumor in nude mice with nano-LDH-LMP1-shRNA1, nanoLDH-LMP1-shRNA2, plasmid LMP1-shRNA1, and LMP1-shRNA2, and found that nano-LDH-LMP1-shRNA1 and nano-LDH-LMP1-shRNA2 treatment significantly inhibited the growth of HNE1-LMP1-tumor *in vivo* and displayed a stronger effect on tumor growth compared with plasmid treatments of LMP1-shRNA1 and LMP1-shRNA2. This indicated that nanomaterials loaded with shRNA plasmid strengthened the effect of RNAi mediated by shRNA plasmid *in vivo* (Figure 4D and E).

To further prove the additional immune stimulatory function of LMP1-shRNA2, we generated the NPC-LMP1 antigen-specific CTLs from NPC-LMP1 cells under the treatment of different sequence-specific shRNAs targeting LMP1 or control gene *in vitro*. We then treated the HNE1-LMP1 tumor-bearing mice by adoptive cell therapy (ACT) using the above antigen specific CTL (including CTL1-3), as shown in Figure 4F. All these antigen-specific CTLs could inhibit the HNE1-LMP1 growth *in vivo*, but the antigen-specific CTLs induced by HNE1-LMP1 cells under LMP1-sh-control and LMP1-shRNA2 administration showed a stronger inhibition on HNE1-LMP1 tumor than that of T cells induced by HNE1-LMP1 cells under LMP1-shRNA1 administration (Figure 4G). These data indicated that the sequence-specific LMP1-shRNA2 but not LMP-1-shRNA1 maintained the immunogenicity of LMP1 antigen and provoked a T cell-mediated anti-tumor response.

## Discussion

Common gene-editing technologies currently in use include RNA interference (RNAi), including small interfering RNAs (siRNAs) and short hairpin RNAs (shRNAs), new generations zinc finger nucleases (ZFNs), transcriptional-activator-like effector nucleases (TALENs), and clusters of regularly spaced short palindromic repeats (CRISPR/Cas9) (25). Among these technologies, nuclease gene-editing technologies, such as TALENs and CRISPR/Cas9, are preferred by most scientists due to their advantages such as short cycle and no species restriction, but their off-target effects are still inevitable (26). RNAi therapy can use the natural molecular machinery of cells to specifically and efficiently knock down targeted genes through the promotion of RNA interference. It is highly active in many important fields and has become a research hotspot especially in the field of cancer therapy (1). Currently, RNAi therapy is more explored by silencing oncogene expression through siRNA or shRNA, and then inhibiting the biological behavior of cancer cells (27). siRNA is unstable and largely depends on an effective *in vivo* delivery system. In contrast, shRNA is superior to siRNA in knockdown genes and can be synthesized continuously after infusion into host cells without continuous administration (28). Here, we provided a sequence-specific shRNA targeting the oncogene LMP1 that not only prevented the oncogenic activation of LMP1 in NPC, but also maintained the immunogenicity of LMP1 protein. Moreover, the LMP1-shRNAs loaded with the nanometer material and the NPC-LMP1-induced CTLs exhibited a stronger tumor growth suppression on NPC xenograft *in vivo*, suggesting that an effective delivery system is important for shRNA delivery.

NPC is a malignancy with EBV infection and has a high incidence in south China (29). However, there is no standard second-line therapy for the treatment of this cancer once it has progressed after chemo- and radiotherapy. A growing number of studies have found that combined immunotherapy may provide a new direction for the treatment of NPC (30-33). Based on these studies, we believed that the sequence-specific RNAi targeting EBV oncogene such as LMP1 would enhance anti-tumor activity with less off-target effects, that the combination or targeted treatment could both suppress the cancer cell biological action and provoke the host immune system to promote anti-tumor immunity in NPC.

LMP1 is a latent phase antigen of EBV presented by 40-60% of tumor cells in NPC (34,35). LMP1 antigen and LMP1-induced tumor-associated antigen (TAA) induces T cell recognition *in vitr*o and *in vivo* (36). In addition, LMP1 is reported to enhance the anti-apoptosis, motility, and invasion of tumor cells and T cell immune suppression in NPC patients (14,15). Therefore, we chose LMP1 as the target gene for RNAi in the treatment of NPC. Our data proved that the NPC-LMP1 cells treated by LMP1-shRNA2 targeting the specific nucleotide locus of LMP1 gene can silence the gene while keep a truncation LMP1 protein expression. We further confirmed that the sequence-specific LMP1-shRNA1 and 2 interfere with the biological action of NPC-LMP1 cells by reducing the ability of NPC cell proliferation, migration, and invasion. More importantly, we have demonstrated that the LMP1-shRNA2 but not 1 kept the immunogenicity of NPC-LMP1 and provoked the antigen-specific CTL anti-tumor immune response *in vitro*. Additionally, both LMP1-shRNA1 and 2 reduced NPC-derived MDSC induction, which stimulates T cell immunity. It has been reported that LMP1 induces immune tolerance in NPC by stimulating MDSC expansion (16). Overall, our *in vitro* data suggested that the sequence-specific LMP1-shRNA2 not only interferes with the oncogenic action of LMP1 gene in NPC but also provokes anti-tumor immunity.

To further verify the anti-tumor effect of LMP1-shRNAs *in vivo*, we established the NPC-LMP1 xenograft tumor model in nude mice and found that the LMP1-shRNA1 and 2 treatments reduced tumor growth in nude mice compared with the control group, though without statistical significance. These findings may suggest that RNAi therapeutic development in NPC must overcome some major challenges, especially in delivery methods. A major limitation of RNAi-based therapeutics is the lack of appropriate delivery systems. Numerous studies have shown that nanoparticles are one of the most widely used delivery systems for RNA (37). Moreover, nanoparticles such as nano-LDH have been shown to have good biocompatibility and can extend the duration of drug action and improve drug stability (38,39). In the present study, we loaded shRNA plasmid to LDH nanoparticles, and found that the nano-LDH-loaded LMP1-shRNAs significantly inhibited tumor growth compared with the corresponding control groups or LMP1-shRNA plasmid alone groups.

Our findings warrant further studies in shRNA therapeutics for clinical application. Furthermore, we did not find any toxicity of the nanoparticle or shRNA plasmid in nude mice (Supplementary Figure S2). Therefore, further optimization of delivery systems to improve clinical efficacy is necessary in the near future. Nanotechnology-based delivery, specifically therapeutic nucleic acids combined with immunotherapy, may eventually improve the therapeutic outcome in NPC patients (40). Finally, the continued development of RNAi therapy with optimized targeting in combination with provocation of anti-tumor immunity may represent a new therapeutic strategy for NPC and other virus-associated cancers.

## Supplementary Material

Click here to view [pdf].
